# Congress of American Physicians and Surgeons

**Published:** 1891-10

**Authors:** 


AT THE Congress of American Physicians and Surgeons, recently held in Washington, D. C., the following Buffalo physicians were present:

Drs. Bartow, Cary, Crego, Howe, Krauss, Park, Parmenter, Potter, Putnam, Snow and Stockton. Papers were presented and read by Drs. Bartow, Howe, Park, Stockton, and Krauss.

Dr. James W. Putnam, of Buffalo, was elected a member of the American Neurological Association, his thesis being entitled, Complete Athetosis with Autopsy.

Dr. Edward B. Angell, of Rochester, a former associate editor of this journal, was also elected to active membership in the American Neurological Association, and had the honor of reading his thesis on Thomsen's Disease, before the association.

